# A Capacitive Humidity Sensor Based on Multi-Wall Carbon Nanotubes (MWCNTs)

**DOI:** 10.3390/s90907431

**Published:** 2009-09-16

**Authors:** Wei-Ping Chen, Zhen-Gang Zhao, Xiao-Wei Liu, Zhong-Xin Zhang, Chun-Guang Suo

**Affiliations:** 1 Department of Microelectronics, Harbin Institute of Technology, Harbin, Heilongjiang, China; 2 Key Laboratory of Micro-Systems and Micro-Structures Manufacturing, Ministry of Education, China

**Keywords:** capacitive sensor, humidity sensor, carbon nanotubes (CNTs), capillary condensation

## Abstract

A new type of capacitive humidity sensor is introduced in this paper. The sensor consists of two plate electrodes coated with MWCNT films and four pieces of isolating medium at the four corners of the sensor. According to capillary condensation, the capacitance signal of the sensor is sensitive to relative humidity (RH), which could be transformed to voltage signal by a capacitance to voltage converter circuit. The sensor is tested using different saturated saline solutions at the ambient temperature of 25 °C, which yielded approximately 11% to 97% RH, respectively. The function of the MWCNT films, the effect of electrode distance, the temperature character and the repeatability of the sensor are discussed in this paper.

## 1. Introduction

Humidity sensors, as a kind of humidity measurement devices, are very important in environmental fields, such as medical applications for human comfort, industrial uses, agriculture, automobiles, etc. [1,2]. Recently, research on humidity sensing materials has focused on macromolecule compounds [3], semiconductor materials [4], porous silicon [5,6], carbon nitride films [7,8] and porous ceramics [9–11], etc. However, the manufacture of humidity sensors with these materials result in less stability and reliability, which confines the development of humidity sensors in practical applications.

Since the discovery of the carbon nanotubes (CNTs) by Iijima in Japan in 1991 [12], more and more researchers have concentrate their attention on this new material which has many special characteristics, such as the large surface-volume ratio, high surface activity, good performance on absorption. CNTs are used in gas sensors [13–15], field emission devices [16,17], electronic switches [18–20] and actuators [21,22].

In recent years, many researchers are gradually concentrating on CNT humidity sensors. For instance, John T. W. Yeow produced a capacitive humidity sensor using CNTs and the capacity increased while the relative humidity (RH) rose [23]. Varghese *et al*. developed a new type of multi-wall carbon nanotubes (MWCNTs) sensor for resistance detection in which the value of the resistance and the RH changed in the same way [24].

Since MWCNTs have porous structures, capillary condensation happens in the MWCNT films which can amplify the capacitance response at low RH due to the higher dielectric constant of water (80) [22]. The CNT-enhanced humidity sensor reported in this paper exploits the capillary condensation effect to realize a sensor. A capacitance to voltage converter circuit is used to transform capacitive signal to voltage signal for detection and the results are discussed.

## 2. Sensor Structure and Theory

### 2.1. Structure and Fabrication of Sensor

**Figure 1.**
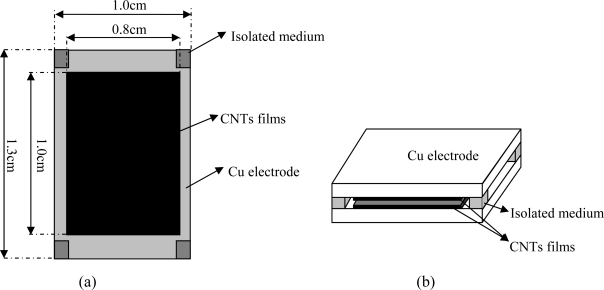
Detailed structure feature (a) and schematic diagram (b) of the capacitive humidity sensor.

The structure of the sensor designed in this paper is shown in Figure 1. Cu was chosen to be the substrate and a film which is mixed with powder of MWCNTs and SiO_2_ is coated by silk screen printing; the thickness of the film is about 10 μm.

The role of SiO_2_ powder is to increase the adhesive properties of the film. The CNTs were first purified by heating at 400 °C. Then they were functionalized by a H_2_SO_4_ and HNH_3_ mixture (v/v = 3:1) at 100 °C for 2 hours to functionalize the CNTs with –OH and −COOH groups, which can make the CNTs hydrophilic and thus increase the sensor sensitivity. Functionalized CNTs were subsequently collected by filtrating and rinsed several times with de-ionized water in order to completely remove the residual acids.

The length and width of the MWCNTs-SiO_2_ film are 10 mm and 8 mm, respectively, and the proportion of both the MWCNTs and SiO_2_ are 50%. Then this principal part of the sensor should be nodulized under vacuum at about 420 °C for 1 hour. Finally, the two Cu electrodes with the MWCNTs-SiO_2_ film on the surface are stuck together with some chemical bond after putting an isolating medium between them on the four corners. The use of an isolating medium is to realize the electrical insulation of the two Cu electrodes.

Two different distances (175 μm and 275 μm) are chosen in this paper to compare their performances in humidity detection. Another sensor without MWCNTs films on the plates is made for comparison to demonstrate the function of the MWCNTs film in RH detection.

**Figure 2.**
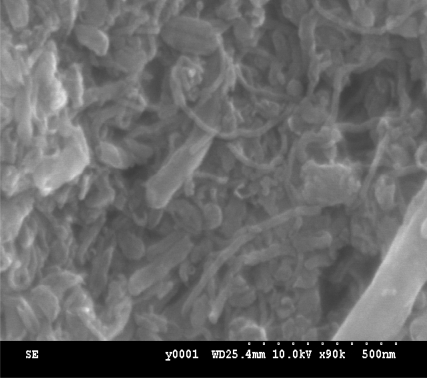
SEM image of MWCNTs-SiO_2_ films.

Figure 2 is the SEM image of MWCNTs-SiO_2_ film, capillary pores are created by the nano-structures of the multi-wall carbon nanotubes. This SEM image indicates that the surface of the film is uniform. Due to the existence of these capillary pores, less vapor molecules from a lower RH level are required to saturate these pores and condense to liquid. Therefore, condensation can occur in these capillary pores earlier at a lower RH level rather than under the normal saturated vapor pressure at high RH or even 100% RH. Because of the condensation of the water modules, the dielectric constant of the sensor, ε’, is changed, and the capacity of the sensor is different at different relative humidity.

### 2.2. Capillary Condensation

The capillary condensation of vapors is the primary method of assessment of structural parameters of materials with pores in the range of 2–100 nm [25]. Vapor condenses to water at a vapor pressure which is lower than the vapor pressure of saturation at a given temperature. Because of the pore structure of CNTs film, a concave surface is produced in the micro-pores. At the same temperature the vapor pressure P corresponding to the concave surface is smaller than the saturated vapor pressure P_0_.

Hence, vapor modules could condense on the concave surface when (P/P_0_) < 1. The condensation always happens from smaller pores to bigger ones which also can be depicted as the stronger the pressure becomes, the bigger the capillary pores where condensation happens are. With a further increase in humidity, the water molecules tend to condense in capillary pores with a radius below the Kelvin radius *r*, which is defined as function (1):

(1) $$r=\frac{-2\gamma V_{L}\;\text{cos}\;\theta }{\mathrm{RT}\;\text{ln}\;(p/p_{0})}$$

*θ*–Contact angle of liquor and wall of capillary pore.
*V_L_*–The molecular volume of liquor.
*γ*–The surface tension of liquor.
*R*–Gas constant (R = 8.314 J × (mol × K)^−1^).
*T*–Absolute temperature (K).
*r*–Kelvin radius of the capillary pores.

According to this equation, for constant *γ* and *θ*, a value of *p/p_0_* will have a value of Kelvin radius *r* associated with it [26]. If the contact angle between the liquor and the wall of concave (*θ*) is constant, we can assume *x* = cos*θ*. For water, the *V_L_* and *γ* are 1.8 × 10^−2^ L/mol and 72.8 mN/m (20 °C), respectively. The temperature (*T*) is about 293K, and the *p/p_0_* is the RH. Thus the values of Kelvin radius are get by function (1), depicted in Table 1. The Kevin radius increases with the relative humidity, and the rate of change (the slope) also increases with relative humidity. In the high RH range, Kevin radius increases dramatically with RH.

**Figure 3.**
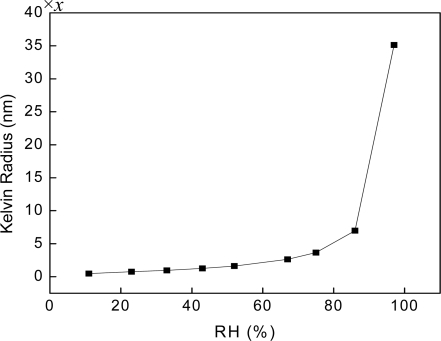
Kelvin radius at different RH.

As shown in Figure 3, at different relative humidity, the Kelvin radius of the capillary pores has notable differences and the value of Kelvin radius grows faster when RH is beyond 50%. It is learned that the higher the relative humidity is, the bigger the Kelvin radius becomes, and the value that Kelvin radius covered at the same relative humidity changed is bigger at high RH.

### 2.3. Detection Circuit

**Figure 4.**
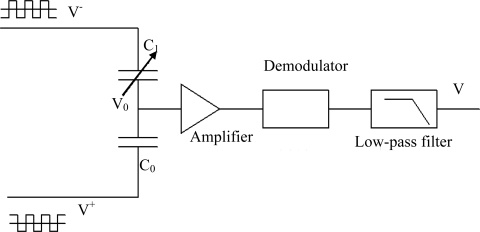
Schematic diagram of the capacitance to voltage converter circuit.

A capacitance to voltage converter circuit is used to detect the capacitive signals to reduce power consumption. The diagrammatic sketch of the circuit is shown in Figure 4. The circuit exports the signal as the form of D.C voltage which changes linearly according to the change of capacity.

V^+^ and V^−^ are square-wave drive signals which have the same amplitude and frequency. The frequency of V^+^ and V^−^ are 270 KHz and the phasic difference of them is 180°. C_1_ is the capacitance of the sensor response to relative humidity. C_0_ is a capacity suited with C_1_. The value of C_0_ can be close to C_1_ through some appropriate regulation and it can be defined as:

(2) $$C_{1}=C_{0}+\Delta C$$

Because the input impedance of the amplifier is very big and the drive frequency of square-wave is high, the influxion of charge from input end of amplifier can be ignored. According to the conservation of charge, the influxion of charge to C_0_ and C_1_ is same, shown in function (3):

(3) $$\frac{V^{-}-V_{0}}{C_{0}+\Delta C}=\frac{V_{0}-V^{+}}{C_{0}}$$

So,

(4) $$V_{0}=\frac{V^{+}C_{0}+V^{+}\Delta C+V^{-}C_{0}}{{2C}_{0}+\Delta C}$$

At the same time, since V^+^ and V^−^ have the same amplitude and frequency, they can be denoted as V^+^ = V+V_t_ and V^−^ = V−V_t_, V is D.C voltage and V_t_ is square-wave signal. Then function (4) can be transferred to:

(5) $$V_{0}=V+V_{t}\frac{\Delta C}{{2C}_{0}+\Delta C}$$

**Figure 5.**
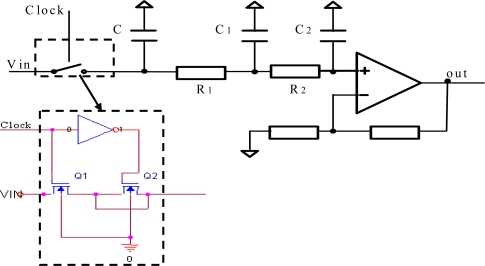
Schematic diagram of switch demodulator and low-pass filter.

If ΔC is much smaller compared with C_0_, the input voltage of amplifier can be denoted as $V_{t}\frac{\Delta C}{{2C}_{0}}$ which is amplified by the amplifier. Then through the demodulator and low-pass filter, a D.C voltage signal is exported. The switch demodulator and low-pass filter are shown in Figure 5, the frequency of clock is the same as square-wave drive signals in Figure 4. The switch K and capacitor C constitute a sample-and-hold circuit, and resistance R_1_, R_2_ and capacitor C_1_, C_2_ constitute a low-pass filter. The cut-off frequency of the low-pass filter is 2 KHz. The switch K is shown in Figure 5, the use of a dummy transistor Q_2_ can cancel clock feedthrough.

**Figure 6.**
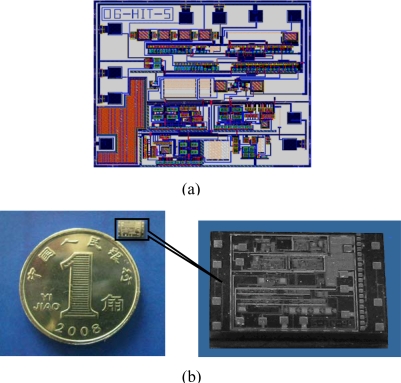
Layout (a) and photograph (b) of the circuit chip.

The readout circuit is simulated by the HSPICE software, whose working voltage is 5 V, and the layout shown in Figure 6(a) is drawn by Cadence. Then the readout circuit is realized with a 0.5 μm double-metal, double-poly CMOS process. A micrograph of the chip is shown in Figure 6(b) and the silicon area is about 3 mm × 4 mm.

## 3. Experiment

Experiments were performed within a closed glass vessel that provides a stable and controllable relative humidity (RH) level at stable temperature. After saline dissolves in the water, the ions of the saline hinder the evaporation of water molecules. If the solution is in a vessel at a stable temperature, the evaporation and condensation will get balance, and the RH of the ambience in the vessel will be fixed. The controlled RH environments are achieved using saturated saline solutions at an ambient temperature of 25 °C which yielded approximately 11% to 97% RH, respectively, depicted in Table 2.

The liquid surfaces in every conical flask stay in the same height, so that the distances between the sensor and the liquor surfaces are constant when the sensors were tested. The sensors are placed close to the liquid surface to guarantee the RH are more precise than theoretical values. To reduce the testing errors, a same readout circuit is used when testing different sensors.

## 4. Results and Discussion

### 4.1. Capacitance Sensitivity to RH

**Figure 7.**
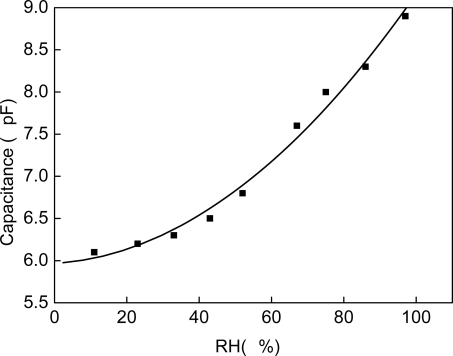
Capacitance sensitivity to RH.

A PM6306 RCL meter is used for testing the capacitance of the sensor, as shown in Figure 7. The capacitance changed from 6.1 pF to 8.9 pF when RH went to 97% RH from 11% RH. Because of the special fabrication of the CNT, capillary condensation occurs in the CNT films where the saturated vapor pressure is lower than on other material’s surface. It causes the transformation from water vapor to liquid. Due to the different relative dielectric constant between water vapor (140 °C, 1.00785) and liquid (close to 80), the capacitance of the sensor changes.

**Figure 8.**
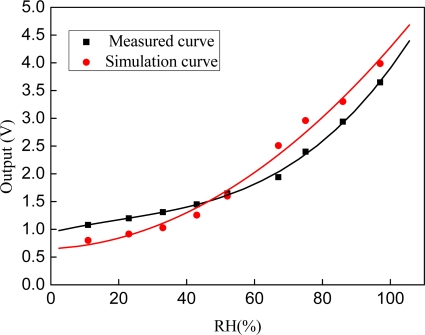
Simulation curve and measured curve of voltage to RH.

The circuit shown as Figure 4 was simulated using the software HSpice and is working properly. As the simulation results, the output voltage of the circuit is linear to capacitance of sensing capacitor, and the slope is 1.137 V/pF. Based on the datum of Figure 7, the output voltage can be calculated out, as Figure 8 (simulation curve) shows.

### 4.2. Output Voltage Sensitivity to RH

The sensor consisted of a sensing capacitor and a readout circuit is tested, the working voltage and power consumption is 5 V and 6.7 mW, respectively. From the testing curve, when the variation of circumstance humidity is from 11% RH to 97% RH, the output voltage catches 3.65 V from 1.08 V. The voltage sensitivity to RH of simulation results is greater than testing result (as seen in Figure 8). This phenomenon is caused by parasitic capacitance (C’). As the integration of the sensor and the circuit is hybrid, parasitic capacitance is inevitable. Probes are used to test the capacitance of the sensor. Before the sensor is connected to the circuit, the static capacitance is about 6.5 pF, and after that the static capacitance changes to 7.7 pF, which means the parasitic capacitance C’ = 1.2 pF.

In order to match the capacitance of the sensor, the fixed capacity C_0_ will change to C_0_’ = C_0_ + C’, and the formula (5) will change to:

(6) $$V_{0}=V+V_{t}\frac{\Delta C}{{2C}_{0}+2C\prime +\Delta C}$$

So the increase of parasitic capacitance can’t convert to voltage by the capacitance to voltage converter circuit directly, but it will reduce the sensitivity of the sensor, as indicated in Figure 8.

The sensor sensitivity (S) is defined as function (7):

(7) $$S=\frac{V_{97\%}-V_{11\%}}{97\%\mathrm{RH}-11\%\mathrm{RH}}\times 100\%$$

where V_97%_ and V_11%_ represent the voltage output of the sensors measured at RH = 97% and RH = 11%, respectively. By the testing datum and function (7), the sensitivity (S) of the sensor is 29.9 mV/%RH.

### 4.3. Comparison of CNTs Sensor and Common Sensor

**Figure 9.**
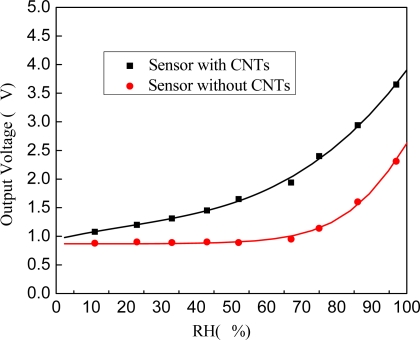
Comparison of the response of the CNT sensor and Cu-plates sensor.

Output voltage of both the CNT-sensor and the Cu-plate sensor are measured and compared at nine different RH points, and the results are depicted in Figure 9. The voltage signal of the Cu-plate sensor change little when the relative humidity is below 67%, but the voltage signal of the sensor with CNTs changed 0.86 V for the same RH change. This phenomenon can be explained by the capillary condensation. The capillary pores in the CNTs film can reduce the vapor pressure P of the vapor, hence, vapor modules could condense on the concave surface at low RH.

### 4.4. Comparison of different distances between two plates

**Figure 10.**
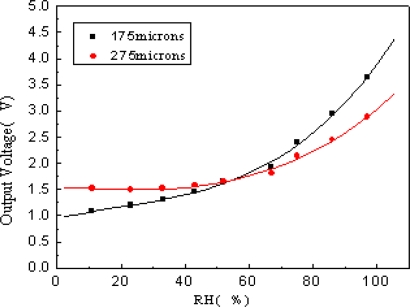
Voltage signal responses of the sensors with different plate distances.

Two sensors with different distances between two plates are tested in room temperature and the results are shown in Figure 10.

The voltage of the narrower one increases faster than the wider one. That is because the capacitance of the narrower one is larger than the wider one, the effect of parasitic capacitance (C') is less evident to the narrower sensor, and then the sensitivity of this sensor is larger than the wider one.

### 4.5. Repeatable Responses of the Sensor

**Figure 11.**
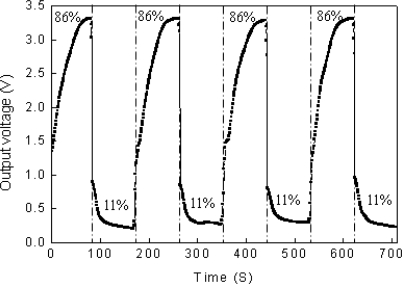
Repeatable responses of the sensor during four cycles between 11% and 86% RH.

The performance of any commercially viable sensor has to be repeatable and reliable. Figure 11 shows reversible changes in the output voltages as the relative humidity is varied cyclically. The experiment is repeated at room temperature at two relative humidity points: 11% (RH) and 86% (RH), and the sensor will stay 1.5 min at each relative humidity alternately. The output voltages of the sensor at 11% RH drift from about 1 V (Figure 8) to 0.3 V (Figure 11) might be caused by the capacitances drift of the adjustable capacitor (C_0_), the parasitic capacitor (C_0_') and the sensor (C_1_).

### 4.6. The Response and Recovery Characters

The response speed of the sensor to humidity is reflected by the response time which is defined as when the RH springs from one value to another, the output of the sensor changes from initial value to 90% of the final value.

**Figure 12.**
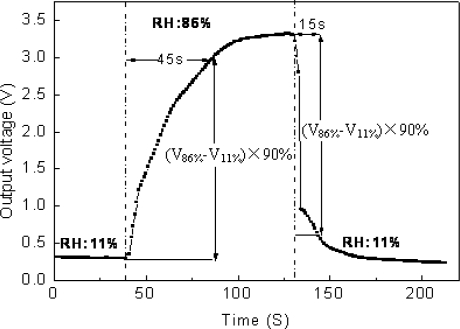
Time of adsorption and desorption procedure.

The response and recovery processes of the sensor can be seen in the test, of which the distance is 175 μm, and this experiment is operated between 11% RH and 86% RH. A Keithley 2000-A digital multimeter is used for testing the output voltage which is gathered by the PC. The response time is 45 s and the recovery time is 15 s according to the processes of adsorption and desorption shown in Figure 12. The time of adsorption procession is longer than the desorption procession, that means capillary condensation will cost much time than evaporation of the water in dry circumstance.

### 4.7. The Temperature Characters of the Sensor

**Figure 13.**
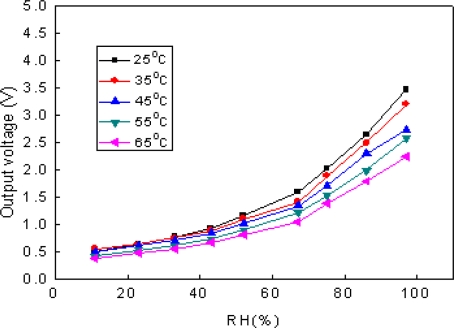
temperature characters of the sensor.

The influence of temperature is a vital characteristic for a sensor. The temperature character of the sensor is measured from 25 °C to 65 °C, and the testing result is shown in Figure 13. The curves indicate that the response of the sensor to RH decreases as the temperature increases. It is because the higher temperature can make the evaporation of water easier to occur, thus the water in the micro-hole in the film decreases and then the response of the sensor reduces.

## 5. Conclusions

A capacitive humidity sensor based on capillary condensation effect is realized in this paper. To reduce the power consumption of the sensor, a capacitance to voltage converter integrated circuit is used. It is shown that the use of MWCNTs can reduce the saturated vapor pressure efficiently and increase the testing range. Making the CNTs hydrophilic and reducing the distance between the two electrodes of the sensor can increase the testing range. For example, the sensor testing range reported by [23] is about 50% to 85% RH, but for the sensor in this paper, the testing range is 11% to 97% RH. Finally, the response speed, the repeatability and the temperature character are analyzed. Although the linearity of the sensor needs more investigation to improve, this kind of sensor has many advantages, such as small size (1.3 cm^2^) and low power consumption (6.7 mW).

## Figures and Tables

**Table 1.** Kelvin radius at different RH.

**RH(%)**	**r(nm)**
11	0.4906*x*
23	0.7369*x*
33	1.095*x*
43	1.283*x*
52	1.656*x*
67	2.704*x*
75	3.764*x*
86	7.180*x*
97	35.55*x*

**Table 2.** RH (%) of different saturated saline solutions.

**Saturated saline solutions**	**15 °C RH(%)**	**20 °C RH(%)**	**25 °C RH(%)**
LiCl	13	12	11
CH_3_COOK	23	23	23
MgCl_2_	33	33	33
K_2_CO_3_	45	44	43
Mg(NO_3_)_2_	53	52	52
CuCl_2_	68	68	67
NaCl	75	75	75
KCl	87	86	86
K_2_SO_4_	97	97	97
